# The Doctor's Pulpit

**Published:** 1888-05-26

**Authors:** 


### THE DOCTOR'S PULPIT.

Constitution Building.—Early Adult Life.

A good many years ago a Yorkshire squire erected a number of cottages on a corner of his estate. Being a not very wise man, he had them first of all built by incompetent stonemasons, who did their work badly; and as a second mistake building operations were discontinued just when the roof was reached. There the cottages remained—roofless, without windows, a melancholy spectacle in a small way of human inconsequence. A few years later, after they had been exposed to the winters' storm and rain, the summers' heat, and the stone-throwing propensities of small boys, the buildings were finished and occupied. What have been the consequences of erecting the cottages badly at first, and of leaving off operations before the work had been carried to completion? Two principal results have followed: first the buildings have cost annually four times as much in repairs as ordinary cottages of their class; and secondly they are already old before they have been built thirty years; older than many cottages in the same neighbourhood which were erected two centuries ago.

This little history is a parable as well as a fact. We have discussed the general principles of human constitution building, and have gone into some details as to infancy and youth. We come now to the time of life which, to the subjects of discourse, is most interesting of all—the period of early adult age, of young manhood and womanhood. This period may be considered the roofing-in and the painting and finishing stage. We will take for granted that parents and schoolmasters have done their work thoroughly and well, and that the young man or woman has reached adult age with a sound and wholesome constitution, both mind and body being in good working order. But the human being at the period of early manhood has not reached his highest stage of development. He may be able to run quickest now, or to clear the tallest gates; but he has not the same degree of energy, mental and physical, and not nearly the same power of endurance, as he should have ten or fifteen years later. The building is, as yet, not completely finished. At the period of adolescence the young man, if not the young woman, is generally supposed to have acquired wings and a certain power of providing for himself. He is then allowed to leave the parent nest and to set up on his own account. His constitution has been so far, to a large extent, built by others. It now falls to his lot to complete the structure, to live in it, and to keep it in repair. First as to the completion. This necessitates two things—attention and avoidance. If a young man is to keep sound and to improve the wholesome body and mind which he finds himself possessed of, he must have the sense to know both what to do and what to leave undone. It does not require the wisdom and experience of a Solomon to know what a young man should do. Many aged people groan because they cannot put "old heads on young shoulders." It would be a great pity if they could. The ways of well-conducted youth are the natural ways for young men.

Ordinarily a youth has to work a definite number of hours daily at this period of life. He is a student, or a clerk, or a mechanic, or something which gives him work to do. So much the better. There is nothing more conducive to health than work. With regard to this, a man should have plenty, but not too much. Very long hours are not good for him. If he begins at six in the morning he should leave off at six in the evening. If he does not begin until nine, he should not go on until nine, but leave off at seven. A man will do more and better work, spending only nine or ten hours a-day at it, than he will by spending twelve or fourteen. One day a week for complete rest is an arrangement which commends itself to every physiologist in the world. If the one day be spent in the old English and Scotch fashion of going twice to church, and strolling in the fields in the interval, the physiological requirements will be as well, or perhaps better, satisfied than in any other way which can be adopted. There is a tendency on the part of many young men to show a little contempt for the Sunday customs of their forefathers, and to adopt an altogether freer and looser tone. We are not preaching religion, but physiology; and our physiological creed in this and every other part of it agrees exactly with the creed of a reasonable religion. Those men are neither scientific nor experienced who encourage young men to substitute a careless and undisciplined way of spending the Sunday for the old-fashioned methods in which we have been brought up. Of course the writer is quite prepared for a superior smile from superior persons; but he is old enough to have tried three or four ways of spending the Sunday, and young enough to hold the broadest possible views on all Sunday questions. As a matter of deliberate scientific opinion he gives his verdict entirely in favour of an orthodox Sunday from a health point of view.

Bodily exercise is the parent of physical strength. It fell to the lot of the writer the other day to examine a batch of young men for insurance. About half of them were clerks, and half did the harder work of the establishment. They were all men of average health and strength, and with one exception were passed on the ordinary terms. But the difference between those who worked with the pen and those who worked with their whole bodies was most striking. The former were paler, flabbier, less prompt and active in movement, altogether of a poorer physique. Of course everybody knows that differences of the kind named are universal, but it is not every day that an opportunity occurs for seeing the contrasts so clearly in large numbers of cases. The examiner envied the workmen their physique, and pitied the clerks for their want of it. But there is no reason whatever why a clerk or a professional man should have a poorer physique than a workman. Dumb-bells are cheap and walking exercise is easy. The man who will practise with the dumb-bells from fifteen to thirty minutes a-day and walk from five to ten miles will not stand second in physical energy and activity to the ordinary workman. Cricket, tennis, football, bicycling—all these are means of bodily salvation to the town man. If a young man will but take his full share of these inspiriting pleasures he will reap an abundant reward.

Intellectual work is almost as essential to perfect bodily health as manual labour. This may seem an over statement of the fact, but it is not so. The ploughman who does not read and think is neither so active nor so strong as his fellow who does both. Just as physical health is essential to the perfection of high and varied mental powers, so is mental activity a general condition of the highest degree of physical health. In the scheme of nature man is a reasoning animal. All his environment is fitted to call into action and to develop his reasoning powers. The less he uses his reason and the more he assimilates himself in action to the lower animals, the poorer are his rewards and successes in the world. That being clearly so, he who desires to build up and maintain a perfect constitution must have an intelligent scheme of self-culture — a scheme of culture which will act as a steady tonic to the brain and nervous system. He must aim at nothing less than a mode of life which will constantly tend to keep in action and to improve his highest mental powers to the last day of his life.

The plan of perfect constitution building would be poorly filled in if no reference were made to moral culture. The man who despises such culture confesses *ipso facto* his mental feebleness and insufficiency. He who has no appreciation of morals is to that extent an idiot, using that word in its proper classical sense. Man is essentially a moral creature. His scheme of morals need not necessarily be conventional or even othodox, but it must be a scheme which he himself recognises. He must admit always the claims of "I ought," or "I ought not." On these fundamental principles he must raise his own scheme, and stand or fall to his own master. But without a moral scheme he cannot be an intellectual man—cannot be truly a man at all.

At the beginning of this paper it was said that in the *completion* of the health structure, for which a young man is himself mainly responsible, *attention and avoidance* were necessary. We have said a good deal about the former; a few words about the latter may fitly conclude. All young men know that certain courses are to be avoided. Some young men avoid those courses. Some try to do so for a time, then fall into them; then justify them. They have to settle these questions in the court of conscience. But they have also to settle them elsewhere. Stories which the present writer could relate from his personal experience would deal with health destroyed, prospects blasted, hearts of parents broken, and character ruined. The doctor in large towns sees more of this than any other man. The diary of many a present physician, even if told with schoolboy simplicity would be more terrible reading than the pages of Samuel Warren. The world is hardened by familiarity with baseness and its physical consequences; but if it were possessed of any imagination at all, it would be filled with horror at the destruction and ruin of human bodies and souls which is going on around us with a dread monotony which knows neither change nor end. If men will despise the preacher, let them not despise the physician. If they doubt the existence of the soul and the future, they cannot question the reality of the body and the present. If they are not afraid of the divine judgment hereafter, let them at least fear the terrible doom which so often falls on offenders here. A life of virtue is a life of health. Self-denial leads to self-development on higher planes. Patient battling against lower lusts ends in assured victory. To one man, and to one only, is life worth living, and that man is he who resolves on nothing less than perfection of body, mind, and soul.

